# Gastric Amyloidosis Presenting as Refractory Dyspepsia: A Diagnostic Challenge

**DOI:** 10.7759/cureus.106655

**Published:** 2026-04-08

**Authors:** Srijeet Ghatak

**Affiliations:** 1 Gastroenterology, Ealing Hospital, Southall, GBR

**Keywords:** diagnosis of dyspepsia, gastroentero-hepatology, git endoscopy, proton pump inhibitor, secondary amyloidosis

## Abstract

Gastric amyloidosis is a rare condition characterized by localized amyloid deposition in the stomach, often mimicking more common gastrointestinal disorders and presenting diagnostic challenges due to nonspecific symptoms and endoscopic findings. A 62-year-old man with six months of dyspepsia unresponsive to proton pump inhibitors underwent endoscopy, which revealed nodular mucosal changes and erosions in the gastric antrum. Biopsy confirmed amyloid deposits with Congo red staining, and systemic involvement was excluded through comprehensive evaluation. The patient improved with symptomatic management. This case underscores the importance of considering isolated gastric amyloidosis in patients with refractory dyspepsia and atypical gastric lesions, with early biopsy and Congo red staining being essential for accurate diagnosis and appropriate care.

## Introduction

This report presents a rare case of isolated gastric amyloidosis manifesting as refractory dyspepsia, a condition that can mimic other gastrointestinal pathologies, making diagnosis particularly challenging. Histopathological confirmation remains the gold standard for diagnosis and should be considered when endoscopic findings are inconclusive or when symptoms persist despite standard therapy [1-6].

## Case presentation

A 62-year-old male with no significant past medical history presented with persistent upper abdominal discomfort and early satiety lasting six months. He denied weight loss, vomiting, hematemesis, melena, or NSAID use. Physical examination and vital signs were unremarkable. Laboratory investigations, including complete blood count, liver and renal function tests, and inflammatory markers, were within normal limits. Serologic testing for Helicobacter pylori was negative.

**Figure 1:**
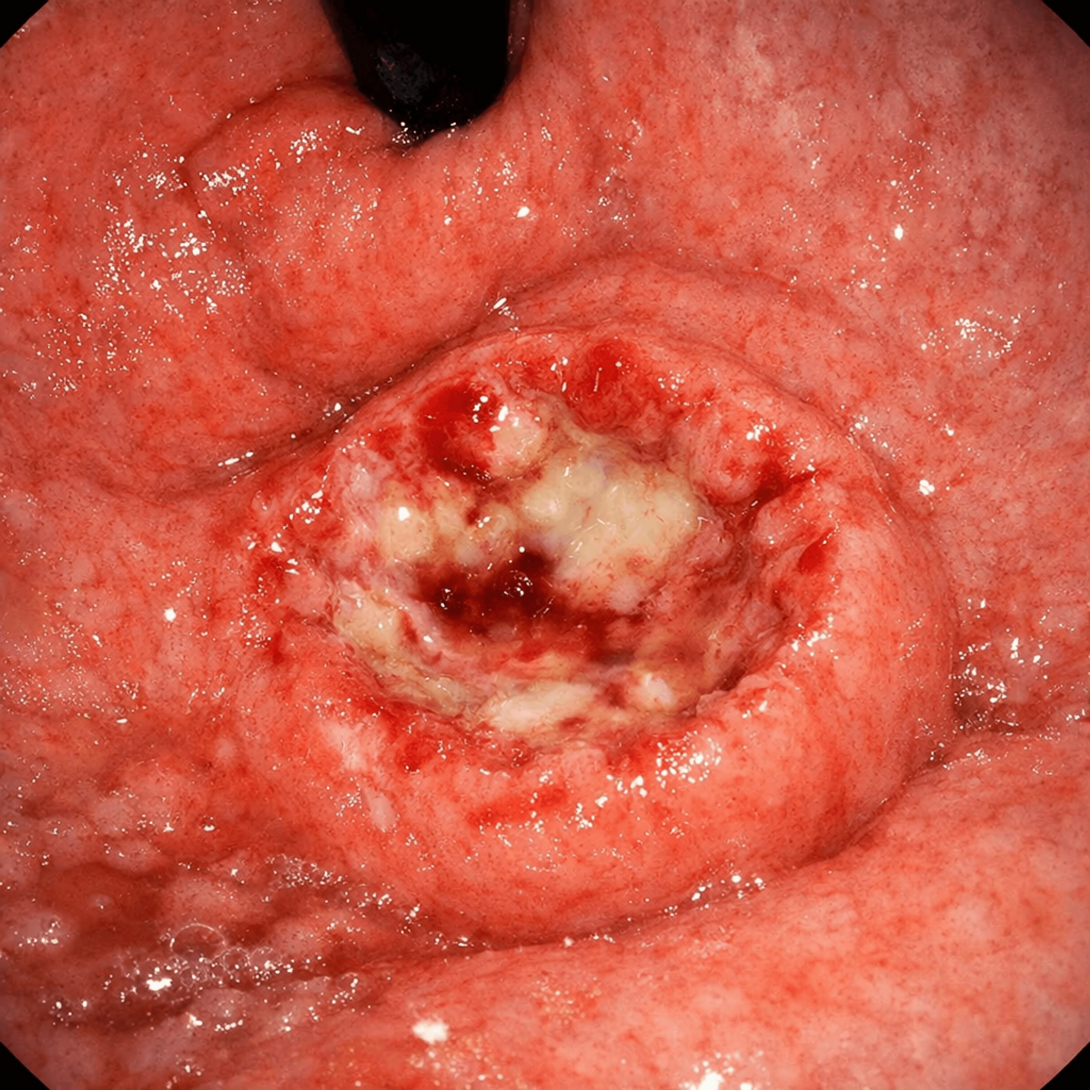
Endoscopy

Esophagogastroduodenoscopy (Figure 1) was performed using a flexible endoscope, advanced under direct visualization through the oropharynx into the esophagus, stomach, and duodenum. The esophageal mucosa appeared normal, with no evidence of inflammation, ulceration, strictures, or varices. Examination of the gastric mucosa revealed multiple nodular, erythematous lesions with superficial erosions, predominantly localized in the gastric antrum and body. The lesions appeared raised with surrounding mucosal redness, suggestive of an inflammatory or infiltrative process. No active bleeding was observed at the time of examination. The duodenal bulb and second part of the duodenum appeared normal, with no mucosal abnormalities noted. Targeted biopsy specimens were obtained from the nodular and erythematous areas in the gastric antrum and body for histopathological evaluation. CLO WAS negative on the biopsy specimen. There were multiple nodular erythematous gastric lesions with superficial erosions, raising suspicion for inflammatory gastritis, infiltrative disease (e.g., amyloidosis), and, less likely, neoplastic process (pending histology).

**Figure 2:**
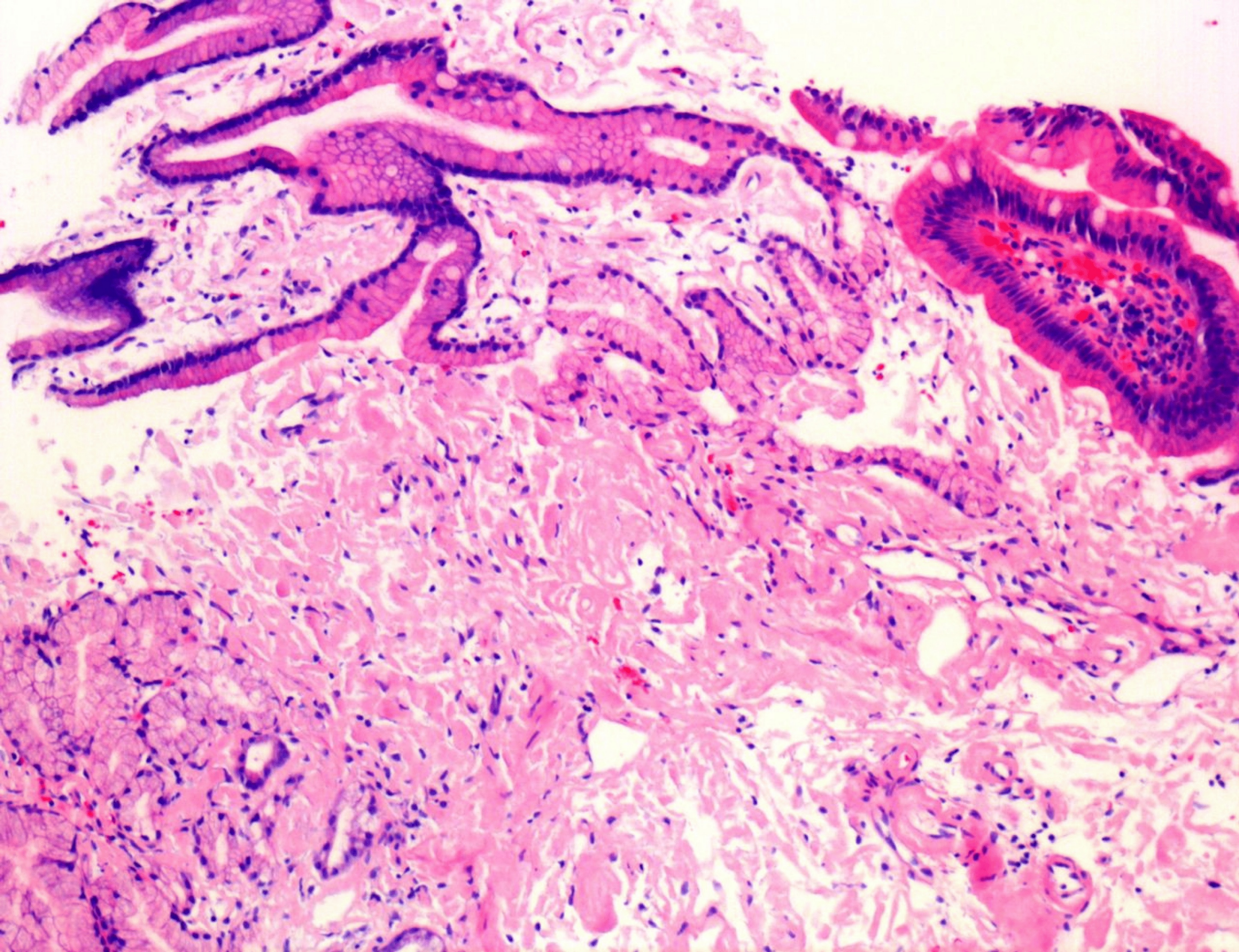
Congo red stain showing amyloid

Histopathology (Figure 2) showed amorphous eosinophilic deposits within the lamina propria. Congo red staining exhibited apple-green birefringence under polarized light, confirming amyloid. Immunohistochemical typing was consistent with AA amyloid.

A comprehensive systemic workup, including serum and urine protein electrophoresis, echocardiography, abdominal fat pad aspiration, and bone marrow biopsy, showed no evidence of systemic amyloidosis.

The patient was managed conservatively with dietary modification and prokinetics. At the six-month follow-up, his symptoms had improved, and no progression was noted on repeat endoscopy.

## Discussion

Gastrointestinal involvement in amyloidosis is typically part of a systemic disease process, most commonly seen in AL (light-chain) or AA (secondary) amyloidosis. Among gastrointestinal sites, the small intestine and colon are more frequently affected, while isolated gastric amyloidosis remains a rare clinical entity [1]. When the stomach is involved, symptoms are often nonspecific, including dyspepsia, epigastric pain, early satiety, nausea, vomiting, or, less commonly, upper gastrointestinal bleeding. Due to this nonspecific presentation, gastric amyloidosis can be easily misdiagnosed as more common gastrointestinal conditions such as gastritis, peptic ulcer disease, or even gastric carcinoma [2].

Endoscopic findings in gastric amyloidosis are highly variable and non-diagnostic on their own. Mucosal changes can range from erythema, friability, erosions, and ulcerations to nodularity or thickened folds, often raising suspicion for malignancy or chronic gastritis [2]. Given this variability, histological evaluation remains essential for diagnosis. Definitive diagnosis relies on obtaining adequate gastric mucosal biopsies followed by Congo red staining. The pathognomonic feature is the demonstration of apple-green birefringence under polarized light, indicating amyloid deposits [3].

Once amyloidosis is confirmed, identifying the amyloid type is critical, as it significantly affects treatment and prognosis. AA amyloidosis, which is associated with chronic inflammatory or infectious conditions, differs from AL amyloidosis, which results from plasma cell dyscrasias or multiple myeloma. Techniques such as immunohistochemistry or mass spectrometry can help distinguish between AA and AL subtypes [4]. Although systemic amyloidosis is more common, isolated gastric involvement, particularly of the AA type, without evidence of systemic disease is rare but has been reported in the literature [5].

Management of gastric amyloidosis depends on the type of amyloid and the extent of systemic involvement. In cases of AA amyloidosis, controlling the underlying inflammatory condition is the primary therapeutic goal. For isolated cases without systemic disease, treatment is usually supportive and focused on symptom control, including dietary modifications and acid suppression therapy [6]. The prognosis of isolated gastric amyloidosis tends to be more favorable than systemic forms, especially when detected early and managed appropriately. Nonetheless, due to its rarity and resemblance to other gastric disorders, clinicians should maintain a high index of suspicion in patients with atypical or refractory gastrointestinal symptoms.

## Conclusions

Isolated gastric amyloidosis, though rare, should be considered in patients with persistent, treatment-resistant dyspepsia and atypical endoscopic findings such as nodularity, erosions, or mucosal friability. As it can mimic gastritis or malignancy, diagnosis requires histopathological confirmation with Congo red staining, which shows apple-green birefringence under polarized light. Once detected, further evaluation is needed to determine the amyloid subtype and exclude systemic involvement, as management and prognosis depend on this distinction. In localized cases without systemic disease, treatment is mainly supportive and the prognosis is generally favorable.

## References

[1] Cowan AJ, Skinner M, Seldin DC (2013). Amyloidosis of the gastrointestinal tract: a 13-year, single-center, referral experience. Haematologica.

[2] Tada S, Iida M, Yao T (1994). Gastrointestinal amyloidosis: radiologic features by chemical types. Radiology.

[3] Picken MM (2010). Amyloidosis-where are we now and where are we heading?. Arch Pathol Lab Med.

[4] Sipe JD, Benson MD, Buxbaum JN, Ikeda S, Merlini G, Saraiva MJ, Westermark P (2014). Nomenclature 2014: Amyloid fibril proteins and clinical classification of the amyloidosis. Amyloid.

[5] Sekijima Y (2014). Recent progress in the understanding and treatment of transthyretin amyloidosis. J Clin Pharm Ther.

[6] Merlini G, Bellotti V (2003). Molecular mechanisms of amyloidosis. N Engl J Med.

